# The Mechanical Representation of Temporal Delays

**DOI:** 10.1038/s41598-017-07289-3

**Published:** 2017-08-09

**Authors:** Raz Leib, Amir Karniel, Ferdinando A. Mussa-Ivaldi

**Affiliations:** 1 Department of Biomedical Engineering, Ben-Gurion University of the Negev, Beer Sheva, Israel; 2 Department of Physiology, Northwestern University, Chicago, Illinois 60611 USA; 3 Rehabilitation Institute of Chicago, Chicago, Illinois 60611 USA

## Abstract

When we knock on a door, we perceive the impact as a collection of simultaneous events, combining sound, sight, and tactile sensation. In reality, information from different modalities but from a single source is flowing inside the brain along different pathways, reaching processing centers at different times. Therefore, interpreting different sensory modalities which seem to occur simultaneously requires information processing that accounts for these different delays. As in a computer-based robotic system, does the brain use some explicit estimation of the time delay, to realign the sensory flows? Or does it compensate for temporal delays by representing them as changes in the body/environment mechanics? Using delayed-state or an approximation for delayed-state manipulations between visual and proprioceptive feedback during a tracking task, we show that tracking errors, grip forces, and learning curves are consistent with predictions of a representation that is based on approximation for delay, refuting an explicit delayed-state representation. Delayed-state representations are based on estimating the time elapsed between the movement commands and their observed consequences. In contrast, an approximation for delay representations result from estimating the instantaneous relation between the expected and observed motion variables, without explicit reference to time.

## Introduction

The propagation of action potentials in an axon and information transfer through chemical synapses is a relatively slow process compared to that of artificial information systems. However, the brain succeeds in producing dexterous movements far superior to any robotic system^1, 2^. Artificial systems handle transmission delays by representing them and then canceling them internally, as in the Smith predictor^3^. In the biological system, it has been suggested that the cerebellum is capable of delay representation^4^.

In a biological system, one may assume that transmission delays change throughout life because of growth, aging, or injury among other factors. Environmental factors also increase the variability of transmission delay. For example, transmission delay differences can explain the flash-lag illusion in which the color and luminance of a flashing stationary object alters the position perception of a moving object when the two are physically aligned^5–7^. Therefore, it is reasonable to assume that the brain must adapt sensory-motor control and information processing to account for these types of variability. Specifically, in this study, we considered the problem of how the brain represents an unexpected but persistent delay between movement commands and their perceived consequences. We examined two possible representations. One was a veridical representation which explicitly accounted for the unexpected time shift using an estimation of the delayed-state. The other was based on a representation which approximate the delay, i.e. the temporal delay was interpreted as a change in the dynamics controlled by the motor command. This second representation effectively resulted in a sensory illusion leading to a reinterpretation of the sensory signals conveying movement state information, e.g., position of a limb and its temporal derivatives. This representation was based on the possibility of approximating a delayed variable as a Taylor series containing the undelayed variable and its successive temporal derivatives.

In earlier studies, it has been shown that when the motor system faces time-dependent force perturbations during arm movements it tends to interpret these forces as state dependent forces^8, 9^. In addition, different computational models suggested that during stick balancing we use an internal model that is based on predicting the sensory consequences of the stick’s movements in order to compensate for the time delay^10, 11^. However, these findings does not exclude the possibility of explicit temporal representations for time delays in different contexts^12^. In a recent study, Farshchiasadeg *et al*. found evidence for the explicit temporal representation in the adaptation of a newly learned pattern of finger movements to externally imposed visuomotor delays^13^.

The brain can compensate for delays between proprioceptive and visual feedback. Introducing a delay between these two sensory modalities during a target tracking task causes a decline in tracking accuracy^14, 15^. Performance, however, improves through adaptation to the delayed environment. Yet, how this adaptation to the delay occurs and how delay is represented by the motor system remains unknown.

As previous studies suggest, internal representations enable the brain to adapt to various conditions such as movement in the presence of external force fields^16^, in manipulation of dynamic objects^17^, and in delayed tracking^14^. Delayed-state based internal representation during target tracking consists of estimating the time elapsed between the movement commands and the observed motion of a cursor. On the other hand, a representation which does not explicitly estimating the time consists of estimating the relation between the expected and observed position, the velocity, and acceleration. Models based on these two different representations predict different trends of adaptation to delay, and the comparison with actual data can indicate which one better describes the representation adopted by the brain.

**Figure 1:**
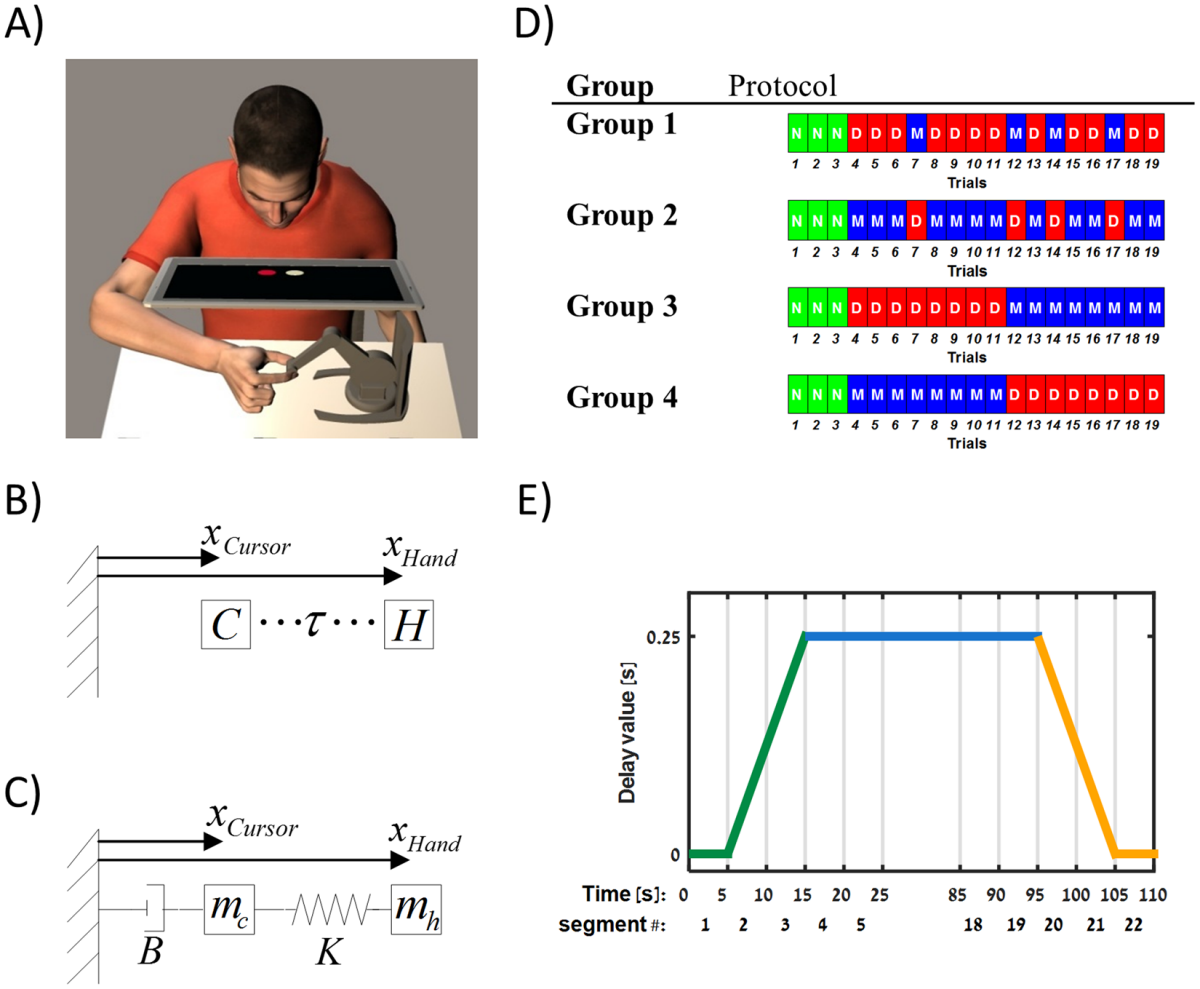
(**A**) Tracking task setup. Participants observed a screen in which two disks representing the position of a moving target (red disk) and the position of the hand (white disk) were presented. While grasping the joint of a robotic device, participants used hand movements to control the white cursor in three possible conditions: Normal tracking (N), Delayed tracking (D), and Mechanical tracking (M). (**B)** Time delay ($\tau$) between cursor position *x*_*Cursor*_ and hand position *x*_*Hand*_. *C* and *H* represent the cursor and the participant’s hand respectively. (**C**) Mechanical approximation to time delay. The cursor position *x*_*Cursor*_ displayed to the participant is the result of an ordinary differential equation based on the hand position *x*_*Hand*_. *B* and *K* represent the damper and spring constants respectively in the mechanical system while *m*_*c*_ and *m*_*h*_ represent the cursor mass and the participant’s hand mass in the mechanical system respectively. (**D**) Experimental Conditions. The order of condition appearance for each group of participants. Each trial is represented using a rectangle with a letter symbol. ‘N’, ‘D’ and ‘M’ represent the Normal, Delay and Mechanical conditions respectively. (**E**) Value of delay between hand and cursor positions during the Delay condition. Delay value increased in the first 15 second of the trial (green line), stayed at a constant value for additional 80 seconds (blue line) and reduced at the end of the trial (orange line). We divided the data of the trial to 22 segments (5 second each).

Following this approach, in a simple tracking task (Fig. 1A), we found evidence that the brain employs a representation which does not explicitly estimate the delayed-state as it learns to compensate for an externally imposed delay. This finding suggests that the neural control system, unlike its artificial counterparts, does not use a time-keeping mechanism such as a biological clock to control and estimate the evolution of ongoing movements. In this sense, instances of apparent time representation reported in the literature^18–20^ may actually reveal a clever use of state representation—namely position and velocity information—instead of an explicit representation of time.

## Results

We used a tracking task where subjects had to control a virtual cursor while chasing after a virtual target (Fig. 1A). Participants observed a monitor where they saw a red disk (the target) moving along a line in a sinusoidal manner and a white cursor which they controlled by moving the endpoint of a small robot. We introduced three types of cursor manipulations: (i) “Normal” condition (N), where the participant’s hand position and cursor position were synchronized, (ii) a “Delay” condition (D), where the cursor position was delayed with respect to the hand position (Fig. 1B), and (iii) a “Mechanical” condition (M), where the cursor position was calculated from the hand position according to the equation of a virtual spring-mass-damper system linking the hand with the cursor (Fig. 1C). The virtual mechanical system was parameterized so as to approximate the behavior of the time delay operator used in the Delay condition (see *Experimental Procedures* for details). We conducted four experiments, all starting with the Normal condition in order to familiarize participants with the task. After that, each group differed in the order of the Delay and Mechanical conditions introduced throughout the experiment (Fig. 1D).

These three conditions require subjects to perform different hand movements. We quantified the tracking success by measuring the spatial difference between the target and the cursor. By this definition, to perfectly track the target in the Normal condition, the participant must move his/her hand exactly like the target, i.e. with the same frequency and amplitude. In the Delay condition, the participant must move his/her hand with the same frequency and amplitude, but with a phase lead of the hand over the target corresponding to the set delay. Thus, compared with the Normal condition, participants had to increase the temporal phase between their hand and target. For success in the Mechanical condition, the participant needs to change hand movement amplitude in addition to generating a phase between the hand and cursor. As we show in the *Kinematic Performance during Delay and Mechanical Conditions* section, in the Mechanical condition, participants needed to move the hand with greater amplitude than the target but with the same frequency. Thus, they needed to create larger and faster movements, increasing the difficulty of the task in kinematic terms.

### Tracking error patterns suggests mechanical system based representation for time delay

**Figure 2:**
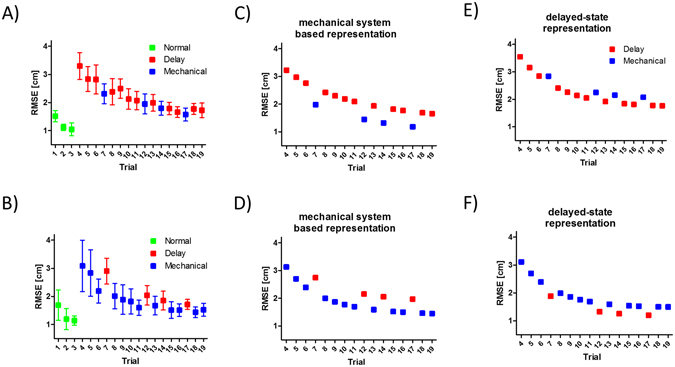
Adaptation to Mechanical and Delay conditions with catch trials of the opposite condition. (**A**) Results of group 1 where participants manipulated the cursor under the Delay condition and experienced unexpected Mechanical catch trials. Squares are the average root mean square error (RMSE) for all participants, and error bars are 95% confidence intervals. (**B**) The same notation as in (**A**) for the results of group 2 where participants manipulated the Mechanical cursor and experienced Delay catch trials. (**C–F**) Tracking errors as predicted by the simulations. Two internal representations were used for these simulations, one which estimates a mechanical system linking hand and cursor position (**C**,**D**), and a second system which estimates the time delay between hand and cursor (**E**,**F**). The two systems were used to control a delayed cursor (trials indicated by red squares) or to control the mass in a mechanical setup representing the cursor (trials indicated by blue squares). Squares represent the average root mean square error (RMSE) between cursor and the tracking target positions. Simulations using the mechanical system based representation best capture the trends exhibited by participants in (**A**,**B**).

After the initial exposure to the tracking task where the hand and cursor were temporally aligned (Normal condition), groups 1 and 2 interacted with a single condition, i.e. either Delay or Mechanical, with occasional catch trials of the second condition, i.e. Mechanical or Delay. As participants in group 1 (Fig. 2A) were exposed consistently to the Delay condition, the sporadic and unexpected presentation of the Mechanical condition produced—if anything—a small, but not statistically significant improvement in their performance (t_140_ = 1.7, *p* = 0.08). However, as participants in group 2 (Fig. 2B) were exposed consistently to the Mechanical condition, the unexpected transition to the Delay condition in catch trials caused a visible and statistically significant worsening in performance (t_140_ = 3.57, *p* < 0.001).

To explain these trends we suggest that tracking accuracy is related to the mismatch between the internal representation of the hand-cursor dynamics and the actual dynamics. Since the error in estimating the cursor position due to visual latencies is similar between the Delay and Mechanical conditions, the difference in tracking performance is related to the error due to inaccurate estimation of the dynamics between hand and cursor. When the internal representation of the hand-cursor dynamics accuratly describes the actual dynamics, we expect a reduction in tracking error. In contrast, when the internal representation does not match the actual dynamics we expect worsening in tracking performance. We used two possible internal representations for the hand-cursor dynamics; (i) the cursor position is delayed after the hand position (delayed-state based representation) and (ii) the cursor position is calculated using the mechanical system in Fig. 1C (mechanical system based representation). For the first representation we suggest that the internal model is based on changing the estimated delay value. For the second representation we suggest an internal model which is based on changing the values of the mechanical elements in a mechanical system that acts effectively like a link between the hand position and the cursor position. We used each internal representation to simulate the hand position while the cursor position was calculated according to the Delay or Mechanical conditions. We simulated 16 tracking trials per simulation, while the condition that was used in each trial was set according to groups 1 or 2 protocols. Thus, for each internal representation we ran the simulation twice. To simulate the decrease in tracking error between trials, we changed the value of the estimated delay or the estimated values of the mechanical elements as a function of trial number so they will be similar to the actual values that was used during the experiments (See *Simulation using Time or Mechanical delay representation in the Methods* section for more details).

We found that the results of groups 1 and 2 are qualitatively consistent with the predictions of a mechanical system based representation (Fig. 2C and D) and are not compatible with the predictions of a delayed-state based representation (Fig. 2E and F). This comparison between learning curves of groups 1 and 2 and the simulation predictions suggests that participants formed a representation that does not explicitly estimate the delay, such as a mechanical system, rather than a temporal based representation, such as delayed-state, of the perturbed environment.

**Figure 3:**
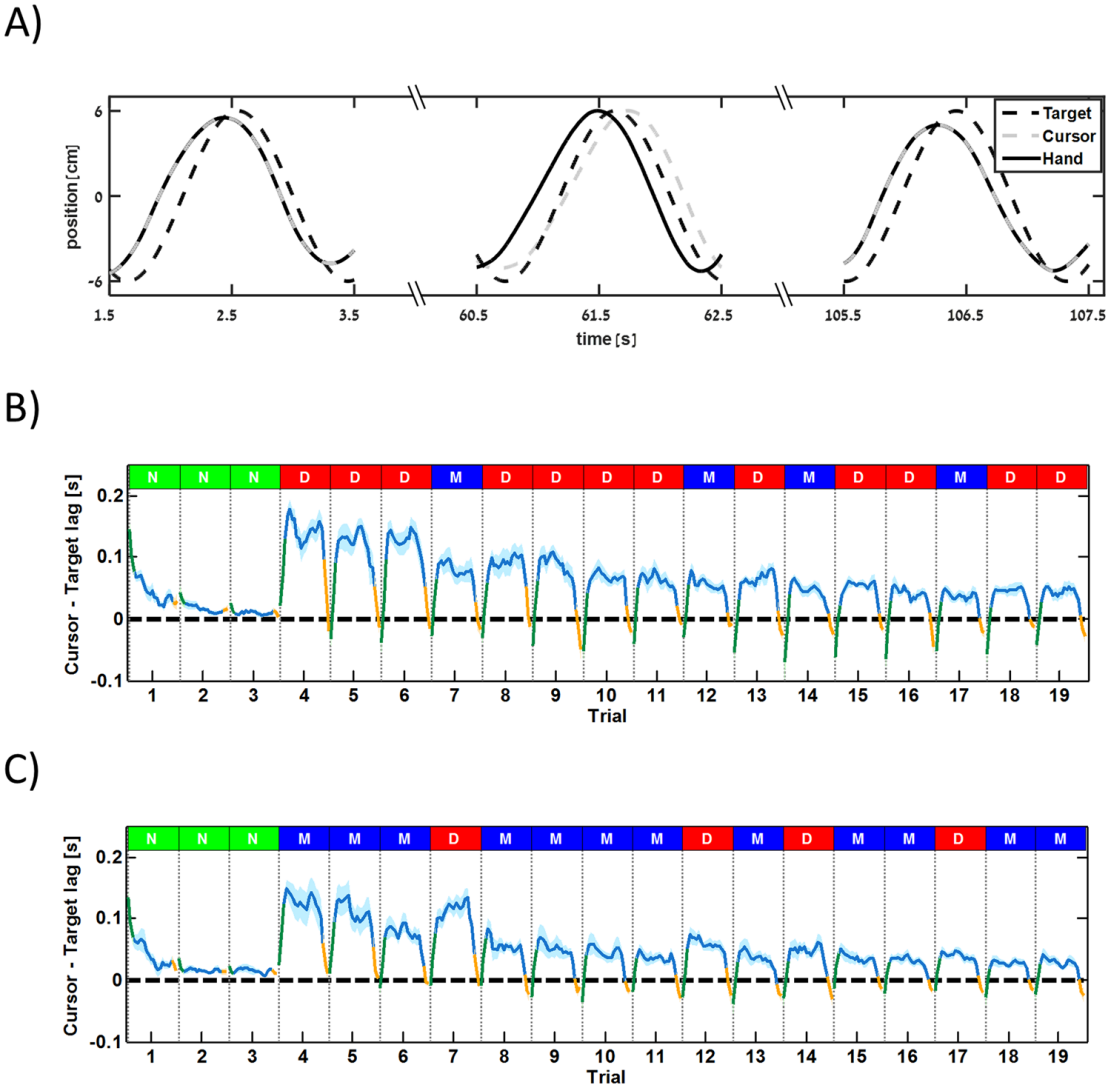
Trial-by-trial analysis of target-cursor lag during different parts of a trial. (**A**) Example for lag changing within a single trial during manipulation under the Delay condition. Without any visual manipulation (seconds 1.5–3.5) the hand (solid black line) and the cursor (dashed gray line) are aligned and usually lead the target (dashed black line) as a consequence of the visual manipulation effect carried over from a previous trial. Introducing delay between hand and cursor (seconds 60.5–62.5) made the participant to try and move his/her hand in such way that it will lead the target so the cursor will be aligned with the target. After the delay was removed, the hand and the cursor realigned which caused the cursor to lead the target (seconds 105.5–107.5). (**B**) Lag value between the cursor in each trial for group 1. Colored lines indicate mean lag value of participants according to the different parts of each trial. The parts are marked with colors according to the delay profile in Fig. 1E. Shaded region represents the STD around the mean. Rectangles with letter symbols represent the condition in each trial according to Fig. 1D. (**C**) The same notation as in (**B**) but for group 2.

To further explore the formation of environment representation we compared the after-effects following tracking under the Mechanical or Delay conditions. To measure the after-effect we calculated the lag between cursor and target signals. An example for temporal lags during the different parts of a single trial is depicted in Fig. 3A. During the first five seconds of the trial where hand and cursor were aligned, the curser slightly led the target. Applying either the Mechanical or Delay visual manipulation made the cursor to lag behind the target throughout most of the trial, causing the participants to try and lead the target with their hand so cursor and target will be aligned. After removing the visual manipulation during the last five seconds of the trial, the cursor led the target since it became aligned again with the participants’ hand which was leading the target. This lead was carried over to the beginning of the next trial which explains why hand and cursor slightly lead the target at the beginning of most trials.

This pattern repeated itself during the trials under the Mechanical or Delay conditions for group 1 (Fig. 3B) and for group 2 (Fig. 3C). To quantify the after-effects in each trial we calculated the difference between the lag during the last period under the visual manipulation and the lag during final period of the trial where there was no visual manipulation. We found that there was no statistically significant effect of the type of perturbation, i.e. Mechanical or Delay, on this difference (for both groups 1 and 2, t_140_ < 1.42, *p* > 0.15) suggesting that participants used the same type of representation in both manipulations.

Using the Laplace transform analysis, we showed that the temporal shift between the hand position and the cursor position during the Delay condition is equal to *τ* = 0.25[sec] while for the Mechanical condition this temporal shift is equal to ${\tan }^{-1}(\frac{2\tau \omega }{2-{\tau }^{2}{\omega }^{2}})/\omega =0.27[\text{sec}]$ (see *Kinematic Performance during Delay and Mechanical Conditions* for more details). Phase analysis results show that although the phase shift is larger during the Mechanical condition, participants were able to adapt to the visual manipulation and quickly readapt to normal tracking at the end of each trial. This analysis suggests increased adaptation difficulty to the Delay condition which caused them to perform worse compared to the Mechanical condition.

**Figure 4:**
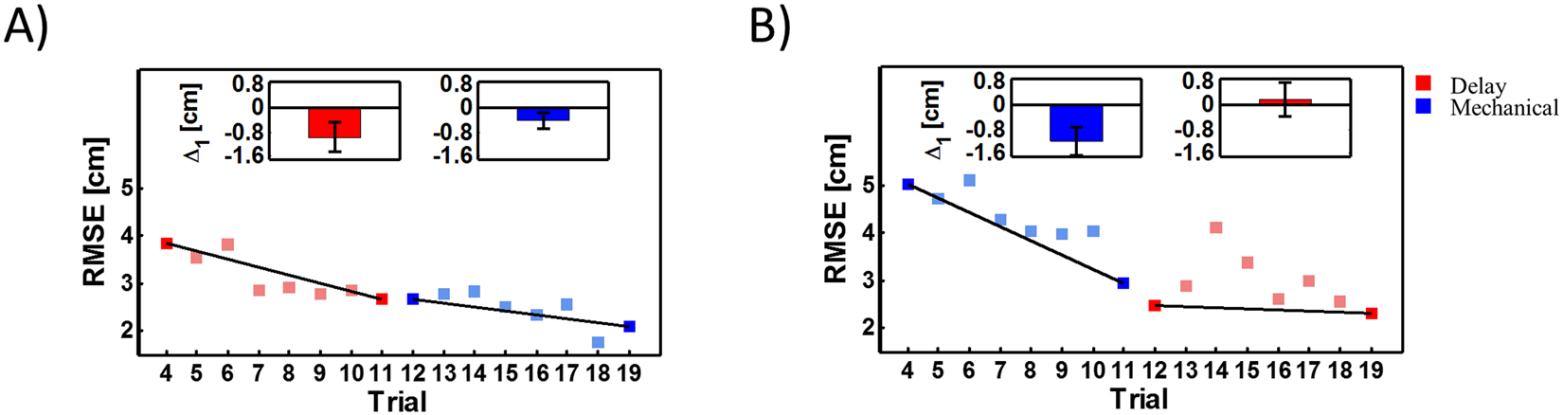
Adaptation while switching between the Mechanical and Delay conditions. (**A**) Performance of a participant from group 3 who switched from the Delay condition to the Mechanical condition. The red squares represent the RMSE of each delay trial, whereas the blue squares represent trials while manipulating the mechanical cursor. For each set, we calculated the difference in RMSE value between the first and last trials (Δ_1_). The inset depicts this matric for all participants. Bars are the mean RMSE difference, and error bars are 95% confidence intervals. (**B**) The same notation as in A for a participant in group 4.

**Figure 5:**
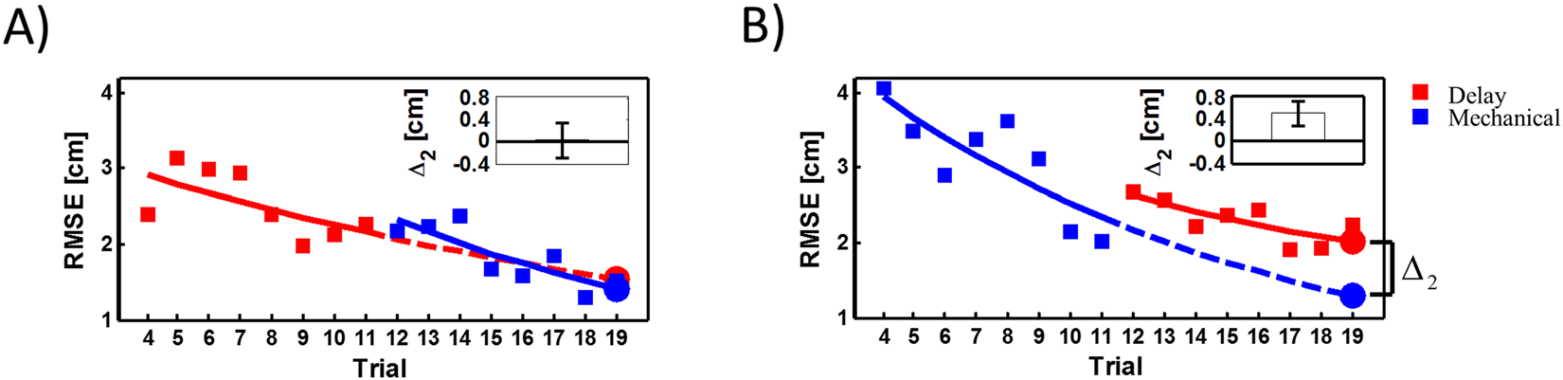
Learning curves during adaptation to the Mechanical and Delay conditions. (**A**) Predicted and actual performance of a participant from group 3. The red squares represent the RMSE of each Delay condition trial, while the blue squares represent the RMSE of Mechanical condition trials. For each set, we fitted learning curves and predicted the error in the 19th trial. Disks with the same color notation represent the predicted error calculated using the learning curves. Δ_2_ is the difference between the predicted error of the mechanical and delayed conditions. The inset depicts the mean Δ_2_ of all participants with error bars representing 95% confidence intervals. (**B)** The same notation as in (**A**) for a participant in group 4.

Figure 4A shows the tracking root mean square error (RMSE) results of one participant from group 3 with a set of trials in the Delay condition followed by a set in the Mechanical condition. The difference between the first and last trial for each condition indicates improvement during both parts of the experiment. Participants that had practiced the Delay condition kept improving their performance after switching to the Mechanical condition (paired t-test, t_14_ = 2.43, *p* = 0.02). When we reversed the order of Mechanical and Delay trials for group 4, we observed on average non-significant improvement as shown in Fig. 4B (paired t-test, t_14_ = 0.68, *p* = 0.5). For groups 3 and 4, we derived learning curves for each participant based on the RMSE of each trial (Fig. 5). We fitted two learning curves for the Mechanical and Delay conditions (overall *R* ^2^ = 0.81 ± 0.07). From these curves we extrapolated the predicted RMSE of the delayed or mechanical tracking. Based on these predictions, we observed that switching from the delayed tracking to the mechanical tracking did not affect the decrease in tracking error (paired t-test, t_14_ = 0.48, *p* = 0.63). However, switching from the mechanical to delayed tracking did cause a significant increase in the RMSE (paired t-test, t_14_ = 4.95, *p* < 0.001). These results suggest that after adapting to the mechanical condition, participants had difficulty switching to the time delay, whereas the opposite order caused less learning interference.

**Figure 6:**
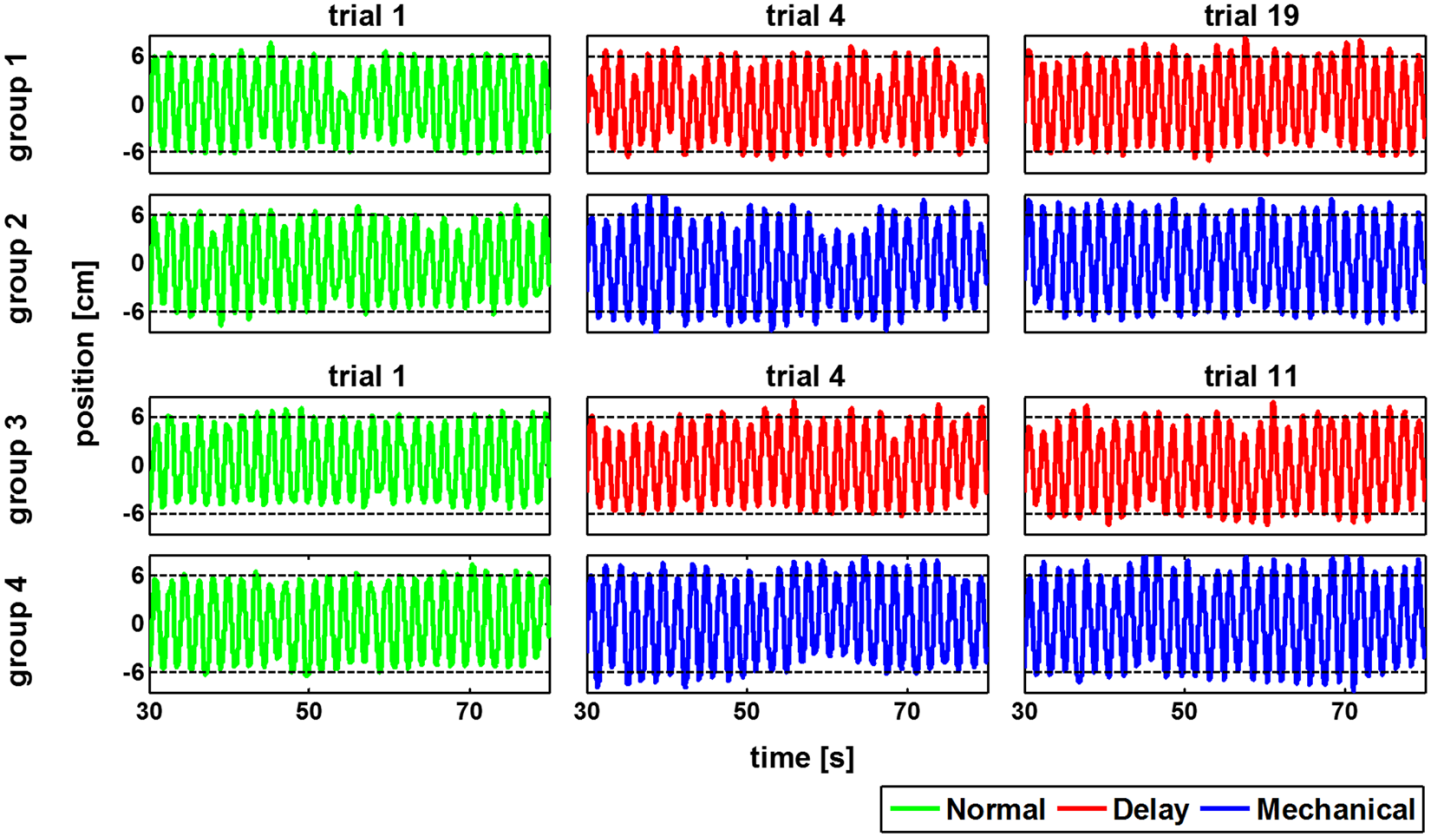
Examples of hand position during tracking under the Normal, Delay and Mechanical conditions. Each raw represents the hand position of one participant from each group. The left column represents the first trial under the Normal condition (green line). The middle column represents the first trial (trial 4) under the Delay (groups 1 and 3, red line) or the Mechanical (groups 2 and 4, blue line) conditions while the right column represent the last trial under the same condition (trial 19 for groups 1 and 2 and trial 11 for groups 3 and 4). Horizontal dashed black lines represent the amplitude of target movement. The examples of participants from groups 2 and 4 suggest that participants increased their hand movement amplitude during adaptation to the Mechanical condition compared to the Normal and Delay conditions.

Figure 6 shows examples for movement amplitude for participants from the four groups during Normal tracking and during the first and last trial under the Delay or Mechanical conditions. Movement amplitude analysis showed that during the Mechanical condition participants moved in larger amplitude (5.8 ± 0.06 cm, mean ± STD) than during the Normal (5.5 ± 0.03) or Delay (5.6 ± 0.06) conditions. Repeated measures ANOVA showed a statistically significant effect of condition on movement amplitude (F_2,98_ = 14.32, *p* < 0.001). Post-hoc analysis showed increased movement amplitude during the Mechanical condition compared with the Normal condition (t_49_ = 4.67, *p* < 0.001) and compared with the Delay condition (t_49_ = 4.4, *p* < 0.001). This result confirmed our analysis using Laplace transform suggesting the participants had to move faster and with a larger amplitude under the Mechanical condition, increasing the difficulty compared with the Delay condition (see *Kinematic Performance during Delay and Mechanical Conditions* in Methods section).

### Grip force modulation supports mechanical approximation for time delay

**Figure 7:**
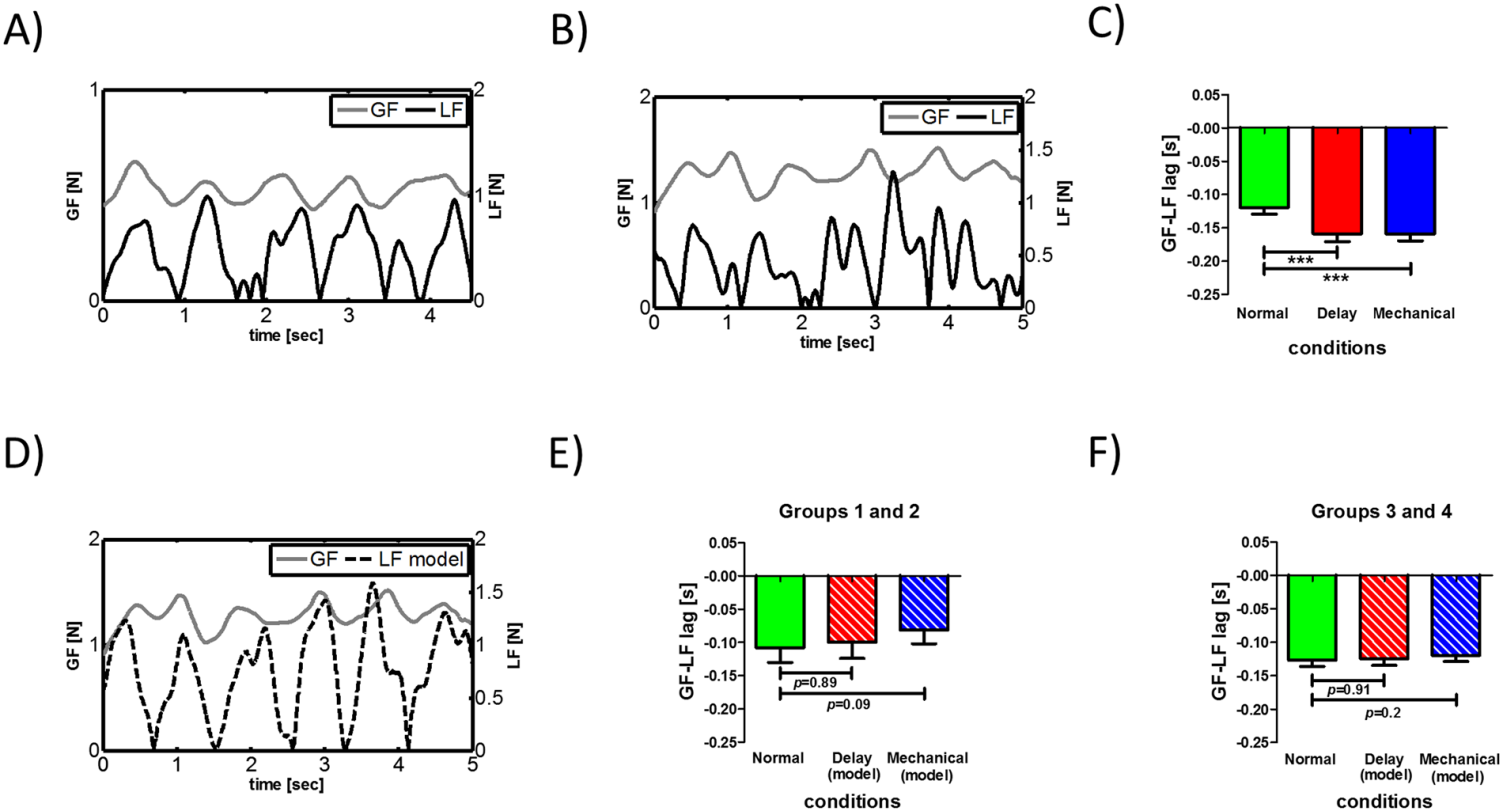
Grip forces (GF) applied during tracking. (**A**) Example for GF during tracking without visual manipulation (Normal condition). In this example the GF (grey line) is slightly leading the load force (LF, marked by a black line). (**B**) Example for GF during tracking under the Delay condition where the phase between GF and LF signals increase significantly. Visual manipulation seems to affect the synchronization between the two signals. (**C**) Group analysis of phase between GF and LF signals. During Normal tracking the GF led the LF (solid green bar). This lead is increased significantly while tracking the target under the Delay (solid red bar) or Mechanical (solid blue bar) conditions. Bars represent mean value across participants and error bars are 95% confidence intervals estimated using t-distribution. ***p < 0.001. (**D**) GF is synchronized with a LF signal (LF model) which is a combination of real inertial forces and virtual forces of the spring in the mechanical model using the same data of the example in (B). (**E**) Group analysis of phase between GF and LF model signals. Phase decreased and resembled the phase during the N condition when we added the virtual force produced by the spring to the actual load force under both the Delay condition (striped red bar) and the Mechanical condition (striped blue bar). Bars represent mean value across participants from groups 1 and 2 and error bars represent 95% confidence estimated using t-distribution. (**F**) Same as in (**E**) but for groups 3 and 4.

During the experiment, both the load force (LF), which was defined using the inertial load (see Methods for more detailed), and applied grip force (GF) exhibited periodic behavior. An example for such behavior during normal tracking is depicted in Fig. 7A. During movement, and similar to previous findings, GF fluctuated according to the LF and slightly led the LF. However, this coupling between the signals changed during tracking under the visual manipulation (Fig. 7B), specifically, the phase between the signals increased significantly. Using cross-correlation between GF and LF, we calculated their temporal phase difference (Fig. 7C). Group analysis showed a statistically significant effect of the visual manipulation (F_2,98_ = 47.52, *p* < 0.001) and post-hoc analysis showed that the phase between the signals during the Mechanical and Delay conditions was larger compared to the phase during the Normal condition (both t_49_ > 7.9, *p* < 0.001). These results are consistent with the results reported in ref. 14.

Since the dynamics of the robot which was held by the participants did not change between the Normal, Delay and Mechanical conditions, we conclude that the visual perturbation caused the change in the grip force-load force coupling. A delayed-state representation suggests that participants will temporally shift their hand position so it will lead the target in order to compensate for the visual manipulation. In such case, the coupling between the load force and grip force remains unchanged since kinematics features of the movement are similar to the Normal condition where hand and cursor are aligned. On the other hand, while considering the mechanical system based representation we can explain the increase in phase between grip force and load force as a change in estimated load force. Based on the works of Sarlegna *et al*. (2010) and Takamuku and Gomi (2015), we explain this change as a result of grip force modulation to a virtual load force that was created by the visual perturbation. By examining the mechanical representation to delay as we suggested here (Fig. 1C), we see that the source for such virtual force may be the spring acting during motion. To test this possibility, we calculated the virtual force using the mean spring constant value we found while calculating the tracking RMSE simulation for groups 1 and 2 (for both groups K = 27 N/m) and added this force to the actual load force the participants experienced during the experiment. When the virtual force signal is added to the actual load force measured during the Mechanical and Delay conditions, the grip force slightly leads the load force, similar to the lead we measured during the Normal condition (see example in Fig. 7D). Group analysis for groups 1 and 2 supported this explanation; the mean phase values depicted in Fig. 7E shows that adding the virtual force to the actual load force reduced the phase between the load force and grip force signals in trials under the Delay or Mechanical conditions, and that the phase was not significantly different from the phase during the Normal condition (F_2,38_ = 2.33, *p* = 0.11). We repeated this procedure for groups 3 and 4. After calculating the simulation for tracking mean RMSE for these two groups in a similar way to groups 1 and 2, we used the fitted stiffness value (for group 3 K = 31 N/m and for group 4 K = 27 N/m) to calculate the virtual force and add it to the actual load force. Group analysis showed the same results similar to groups 1 and 2 (Fig. 7F). The phase between GF and LF, assembled from the real and the virtual signals, was reduced and was not significantly different from the phase during the Normal condition (F_2,38_ = 1.22, *p* = 0.308).

**Figure 8:**
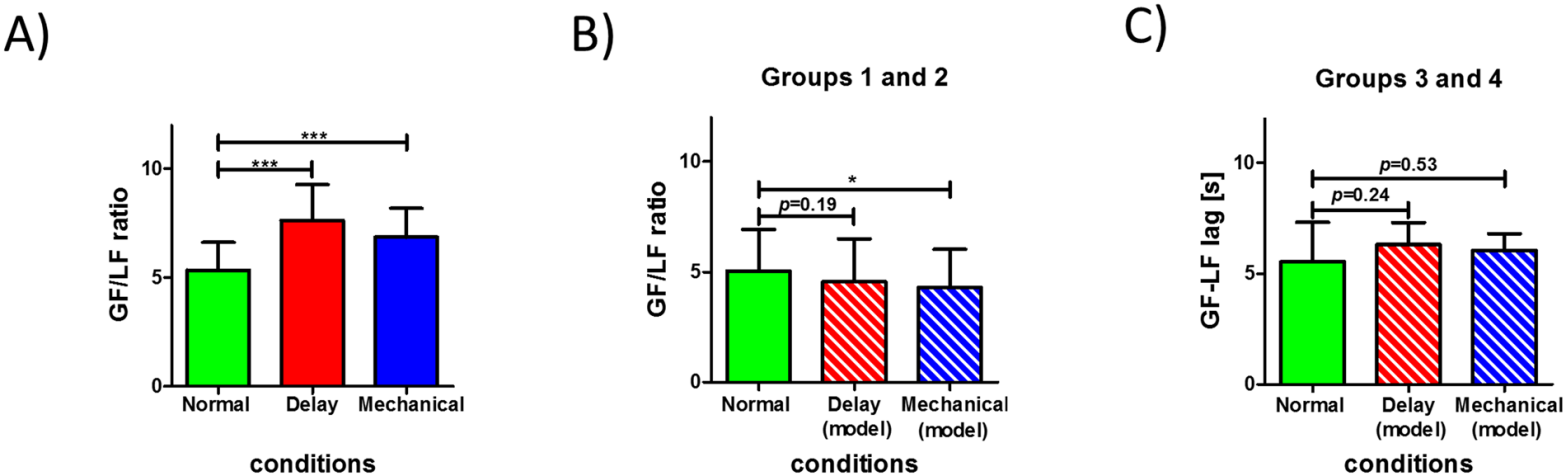
(**A**) Group analysis of Grip force scaling. Increase in value of the ratio between Grip force (GF) and Load force (LF) during tracking under the Delay (solid red bar) or Mechanical (solid blue bar) conditions compared with the Normal condition (solid green bar). (**B**) Adding the virtual force to the real load force decrease the value of the GF/LF for the Delay (striped red bar) and Mechanical (striped blue bar) conditions to values that resembles the value for the Normal condition (solid green bar). Bars represent mean value across participants for groups 1 and 2. (**C**) Same as in (**B**) but for groups 3 and 4. Error bars are 95% confidence intervals estimated using t-distribution. *p < 0.05, ***p < 0.001.

In addition to the phase between grip and load forces we compared the grip force scaling during the three conditions. Analysis of the mean grip force to load force ratio (GF/LF) showed that the visual manipulation affected this metric (F_2,98_ = 21.158, p < 0.001, Fig. 8A). Specifically, during the Delay and Mechanical conditions the GF/LF ratio increased compared to the Normal condition (for the Delay condition t_49_ = 5.71, *p* < 0.001 and for the Mechanical condition t_49_ = 4.7, *p* < 0.001). As for the change in grip force-load force phase, we used the mechanical approximation for time delay to explain this increase in grip force. By adding the virtual force generated by the spring of the mechanical system to the real load force, the GF/LF ratio is decreased for the Delay and Mechanical conditions. We found this reduction in ratio value for groups 1 and 2 under the Delay and Mechanical conditions, although the values are significantly different from the ratio value under the Normal condition (F_2,38_ = 4.487, p = 0.018). Post-hoc analysis showed that there is a difference in GF/LF values between the Mechanical and the Normal conditions (t_19_ = 2.72 *p* = 0.02, Fig. 8B), but not between the Delay and the Normal conditions (t_19_ = 1.58 *p* = 0.19). For groups 3 and 4 we found no statistically significant difference between GF/LF ratio values under the three conditions while calculating the ratio with the added virtual force (F_2,58_ = 0.507, *p* = 0.6, Fig. 8C).

## Discussion

In these experiments we have found experimental evidence and theoretical support for the hypothesis that in a tracking task the central nervous system compensates a visuomotor delay by forming a dynamical model, which accounts for the delay not as an independent temporal shift but as the outcome of a mechanical perturbation. Tracking errors, adaptation, and grip force results reported here all support a mechanical system based representation as the underlying mechanism for the compensation of time delay within a sensory-motor control loop. Our results also provide evidence against explicit temporal representation. This idea of mechanical system based representation for time is consistent and can explain recent results regarding time perception^21–23^.

Using tracking error analysis we showed that tracking performance decreased during catch trials when switching from Mechanical condition to Delay condition and improved when switching from Delay to Mechanical (Groups 1 and 2). In addition, we show that participants kept improving their performance when switching between tracking under the Delay condition to the Mechanical condition but not when switching from the Mechanical condition to the Delay condition (Groups 3 and 4). We propose that the explanation for these trends is based on the mismatch between the internal representation of the hand-cursor dynamics and the actual dynamics. We can describe the formation of the internal representation as the result of interaction between feed-forward and feedback control mechanisms. The feed-forward control mechanism is responsible for generating hand motion according to the predictions about the cursor position, while the feedback mechanism is responsible for adjusting the internal representation of dynamics properties, such as the delay or the spring, damper and mass. We suggest that for both conditions used in this study participants formed a representation which is based on an approximation for delay for the visual perturbation. As a result any additional error under the Delay condition is generated due to inaccurate prediction of the actual cursor trajectory.

Visual latencies may also explain some of the tracking errors we observed in this study. During the Normal condition, where the hand and the cursor were aligned, we observed a temporal lag between the target and hand/cursor positions at initial stage of the experiment (trial 1) which caused an increase in tracking error. The existance of this lag suggest inaccurate estimation of both the target and cursor positions which can be accounted to visual latencies. Different studies showed the role of visual latancies in illusion and how they vary as a function of basic visual parameters like luminance^5–7^. In our setup the display of the virtual environment was similar within the three conditions (Normal, Delay and Mechanical), that is, the colors and luminance of the cursor and target disks were similar. This means that the visual delay existed within conditions and the changes in tracking accuracy between conditions reported in this work are due to the visual manipulation we introduced.

Similar to previous findings^14, 24–27^, we saw an adaptation pattern to the perturbed visual feedback. Interestingly, in some of these studies participants reported illusions regarding changes of their arm mechanical properties^24, 28, 29^. Visual delayed feedback can also create perceptual illusions regarding external mechanical properties^30, 31^. We suggest that these illusions support the idea that participants adapted to visual delayed feedback by constructing a mechanical representation of delay which creates apparent dynamics changes and thus creates the illusion.

We suggest that a mechanical system based representation, and in particular a mechanical system that is driven from a Taylor series approximation for time delay, can be incorporated in existing models to better explain behavioral results following adaptation to delayed visual feedback. Models that rely on explicit time delay estimation, such as in refs 32 and 33, may be improved by considering mechanical system based alternatives to delay estimation. In addition, considering a mechanical system as a representation to delay may give a simple and intuitive interpretation to the effect of delay on various motor activities^34^. However, as discussed in ref. 35, using a Taylor approximation for delay is not applicable for some systems as this may cause instability.

Our research leaves a few issues to be clarified. Although moving at a higher speed for a longer distance, participants were still able to track more accurately during the Mechanical condition. This is not in contrast with the known speed–accuracy tradeoff^36, 37^ since we compared two different conditions rather than the same condition at different speeds. In addition, the need to move at higher speed and amplitude may or may not increase the physiological difficulty to perform the tracking during the Mechanical condition. Nevertheless, participants did not perform worse but instead improved their performance compared to the Delay condition.

In addition, to further establish the results presented here we can try and generalize the idea of mechanical system based representation for time delay to different tracking tasks with different parameters. Instead of a single sinusoidal tracking, a more complex and less predictable trajectory may be used for the target position. Another possibility is to continuously change the delay value between hand and the cursor representing it which will incorporate both steady state tracking, as in this study, and transit response to the delay. Since the perturbation between cursor and hand will be time dependent, we assume that such manipulations will be difficult to adapt to, see for example ref. 8. In such case we predict that participants will have to go through intensive training in order to reduce the tracking error.

In a previous study, we found that the brain can successfully compensate for delayed force perturbations; however, we were not able to distinguish whether this is an outcome of forming delayed-state representation or alternative representation^38^. By claiming that humans can represent a time-dependent distortion via an equivalent mechanical system, we indirectly imply that humans can assess and construct such representation of dynamics via visual information^39, 40^. Using visual sensory-motor coupling, it is suggested that the motor system constructs a mechanical system functionally equivalent to the external environment. This concept has received growing attention in studies of infants representing forces acting upon objects^41^, of adults representing object load force^14^, sensation of force during delayed tracking^29^ and of attempts to estimate the content inside a box^42^. In our study, we have found that participants used a mechanical system based representation rather than a delayed-state based representation for time delays up to 250 milliseconds and suggested that such delays can be approximated using a representation that is associated with a mechanical system. Conceptually, this representation shares the same limits of a truncated Taylor expansion by successive time derivatives, for example, instability of the Taylor series expansion^35^. We, therefore, expect that this approach would fail for sufficiently large time delays^24, 35^. At that point, the only option available to the brain would be the explicit estimate of a temporal interval. Indeed, previous studies showed that during tracking with high values of visual delay participants moved their finger in complex rhythms while the required tracking oscillation appeared intermittently^32, 43^. An important goal for future studies would be to investigate where this break-up takes place under normal conditions by following a variety of neurological disorders that impair the ability to represent the state of limb motion.

## Methods

### Experimental Procedures

Fifty participants (28 males, 22 females between 22 to 29 years of age) divided into four groups participated in one of four experiments (Group 1: ten participants, Group 2: ten participants, Group 3: fifteen participants, Group 4: fifteen participants). All participants gave their informed consent by signing the informed consent form as stipulated by the Institutional Helsinki Committee, Beer-Sheva, Israel. The experimental protocol was approved by the same local ethics committee and the methods were carried out in accordance with the relevant guidelines and regulations. The participants were seated and used their dominant hand to hold the handle of a Phantom desktop haptic device (SensAble Technologies), which was used to record real-time hand motions and to create a force channel to constrain the motion to one dimension. An ATI Nano 17 force sensor was mounted on the handle of the Phantom device to record the applied grip forces. The signals from the haptic device and the force sensor were sampled at 400 Hz. In front of each participant, we placed a computer screen rotated by 30 degrees in such a way that the participant could not see their hand. The computer screen displayed a target (a red disk) moving in a sinusoidal manner ${x}_{{Target}}(t)={A}_{T}\cdot \,\sin (\omega t)$ and a cursor (a white disk), the position of which was calculated according to the current hand position under three different conditions: (i) Normal condition (N)—The cursor position tracked the current hand position ${x}_{Cursor}(t)={x}_{Hand}(t)$, (ii) Delay condition (D)—The cursor position lagged the hand position by 250ms (Fig. 1B), i.e. ${x}_{Cursor}(t+0.25)={x}_{Hand}(t)$, and (iii) Mechanical condition (M)—The cursor position was derived from a simulation of a spring-mass-damper mechanical system (Fig. 1C), described by the ordinary differential equation ${x}_{Hand}(t)={x}_{Cursor}(t)+\frac{B}{K}\cdot {\dot{x}}_{Cursor}(t)+\frac{M}{K}\cdot {\ddot{x}}_{Cursor}(t)$. In all three conditions, due to the sampling process and computations of the visual feedback, an additional delay of 1–2 milliseconds was introduced between the participants hand position and the cursor. None of the participants had previous experience with the tracking task. Therefore, each experiment started with three trials in the Normal condition to familiarize participants with the system and task, followed by 16 trials where the cursor position was calculated according to the Delay or Mechanical conditions. The difference between experiments, i.e. between the groups of participants, was the number and order of each condition appearance as shown in Fig. 1D. The order of the conditions was constant for all the participants within each group. Participants were not informed about the visual manipulation or changes in conditions throughout the experiment.

For all four experiments, we set the target to move with an amplitude of 6 cm at a frequency of 0.556 Hz, i.e. ${A}_{T}=6[cm]$ and $\omega =2\cdot \pi \cdot 0.556[rad/\sec ]$. Each trial lasted 110 seconds. In the N condition, the cursor position was aligned with the hand position for the entire 110 seconds. In the Delay condition, we gradually increased the delay to 250 milliseconds (Fig. 1E); for the first five seconds, the cursor position was aligned with the hand position. Afterwards, the delay increased linearly to the value of 0.25 [sec] over ten seconds. This value of delay was maintained for 80 seconds and then gradually decreased back to 0 in ten seconds, remaining at this value for another 5 seconds. In the Mechanical condition, we used the same timing and technique of increasing and decreasing the values of the ratios *M*/*K* and *B*/*K* as explained below.

### Mechanical Approximation to Delay

In this study, we suggest that time delay is represented using a mechanical equivalence derived from the Taylor series expansion. By truncating the Taylor series approximation after its third term, one obtains:

1 $$x(t+\tau )\cong x(t)+\tau \cdot \dot{x}(t)+\frac{{\tau }^{2}}{2}\ddot{x}(t)$$

Applying this approximation to the delayed cursor position as in the Delay condition, we get:

2 $${x}_{Hand}(t)={x}_{Cursor}(t+\tau )\cong {x}_{Cursor}(t)+\tau \cdot {\dot{x}}_{Cursor}(t)+\frac{{\tau }^{2}}{2}{\ddot{x}}_{Cursor}(t)$$

The mechanical equivalence we are proposing here is presented in Fig. 1C. This environment sets the following dynamics derived from Newton’s Second Law:

3 $${x}_{Hand}(t)={x}_{Cursor}(t)+\frac{B}{K}\cdot {\dot{x}}_{Cursor}(t)+\frac{M}{K}\cdot {\ddot{x}}_{Cursor}(t)$$

By setting the equalities $\frac{M}{K}=\frac{{\tau }^{2}}{2}$ and $\frac{B}{K}=\tau$ we get the mechanical approximation for the time delay operator. This approximation implies that in order to build an internal representation of the dynamics between the hand and the cursor the system does not estimate the delayed-state, but instead tries to approximate the corresponding mechanical parameters.

### Data analysis

To analyze the tracking performance of each participant we calculated the tracking error between the target and cursor for each trial using the Root Mean Square Error, $RMSE=\sqrt{\frac{1}{N}\sum _{i=1}^{N}{({x}_{{Target}}[i]-{x}_{Cursor}[i])}^{2}}$, where *N* is the total number of samples for each trial. We calculated the RMSE of each trial during the 80 seconds where the values of the delay or the ratios of the mechanical system reached their maximum values (blue line in Fig. 1E). For the Normal condition, we calculated the RMSE for each trial during the same 80 seconds. For groups 3 and 4 we derived the learning curves for each participant based on the RMSE of each trial by fitting a double exponential function (using least squares method).

In addition, for groups 1 and 2 we calculated the time lag between cursor and target positions using cross-correlation between the signals. We divided each trial to 22 segments, each lasting 5 seconds, and for each segment we extracted the lag value for which cross-correlation was at maximum value. To check how removing the Mechanical or Delay visual manipulation affected this lag, namely the after-effect, we calculated the difference between the lag of the last segment with the manipulation (segment 19 in Fig. 1E) and the last segment of the trial where hand and cursor were realigned (segment 22 in Fig. 1E).

The motor task in our experiment includes rhythmic hand motion while holding a rigid object (Fig. 1A). In such case and since no other forces acted on the participants’ hand in the direction of motion, the load force acting on the participants’ hand is defined as the object acceleration multiplied by its mass (inertial load). To calculate the robot’s arm acceleration we differentiate the robot’s position signal. This signal was then filtered using fourth-order Butterworth filter (12 Hz cutoff frequency with no phase shift using *filtfilt* function in Mathworks® Matlab). In a similar way to calculating the lag between cursor and target, we calculated the lag between the load force and grip force signal for each time segment (overall 22 segments) in each trial. For each trial, we averaged the lag values of segments where delay or mechanical ratios were at their maximum value (segments 4 to 19, see Fig. 1E). To test the GF scaling, we again used the grip force and load signals and calculated the mean GF to mean LF ratio (GF/LF) for segments 4 to 19 for each trial.

### Statistical analysis

To analyze the effect of switching between visual manipulations (Mechanical or Delay) on tracking RMSE values for groups 1 and 2 we used repeated measures analysis using a mixed model with trial number (16 trials), perturbation (Mechanical or Delay) and their interaction as fixed effects. Trial number was also used as a random effect. We used the same analysis to check the effect of switching between visual manipulations on the different time lag between cursor and target for trials of groups 1 and 2.

Changes in lag between grip force and load force signals and GF to LF ratio were examined using one-way repeated measures ANOVA with condition (Normal, Mechanical and Delay) as within factor. We performed post-hoc analysis using Bonferroni correction for multiple comparisons. Statistical significance was determined at the 0.05 threshold in all tests.

### Simulation using Time or Mechanical delay representation

The goal of these simulations was to determine which of the two representations—delayed-state or mechanical system—was used to generate a feed-forward command that can account for the trends exhibited during the catch trials of groups 1 and 2. We postulate two internal representations to describe the dynamics between hand and cursor positions while tracking the target under the Mechanical and Delay visual manipulations. For both types of representations, we assumed ideal estimation of the target trajectory, i.e. ${x}_{Cursor}^{Desired}(t)={x}_{{Target}}(t)={A}_{T}\,\sin (\omega t)$. We assumed that the participants can accurately estimate the amplitude and frequency of the target motion. During the experiment, the movement amplitude and frequency were similar to the values we used in the experiment (Fig. 5). Thus, the tracking errors are mostly the outcome of inaccurate estimation of the phase between the hand and the target under the Delay or Mechanical conditions.

Using delayed-state representation for delay, the desired hand trajectory can be calculated using the estimation of the delay ($\tilde{\tau }$) between the hand and cursor:

4 $${x}_{Hand}(t)={A}_{T}\,\sin (\omega (t+\tilde{\tau }))$$

For the case of Normal tracking, i.e. there is alignment between the hand and cursor position, $\tilde{\tau }$ is equal to zero. Under the Delay or Mechanical conditions, the value of the estimated delay will change to generate the desired cursor trajectory. For example, if estimated delay remains at zero value during tracking under the Delay condition, the hand will be aligned with the target but the cursor will not be aligned with the target creating tracking errors. To simulate the changes in the estimated delay value, we assumed an exponential increase of the estimated delay value between consecutive trials, $\tilde{\tau }={a}_{1}+{a}_{2}\cdot \exp (i/{a}_{3})$, where *i* indicates the trial number, and *a*_1_, *a*_2_, *a*_3_ are constants. The constants were found separately for groups 1 and 2 so each simulation’s tracking RMSE pattern will be identical as possible to the mean RMSE pattern of each group (Fig. 2). The tracking RMSE pattern which we tried to mimic included only RMSE values of the dominant condition used in each group protocol (without the catch trials), i.e. for group 1 the Delay condition and for group 2 the Mechanical condition. After finding the delay estimation function we simulated the entire experiment protocol by introducing catch trials according to the experimental protocol of each group (Fig. 1D) and calculated the simulation’s tracking RMSE during these catch trials. We tested the model’s RMSE prediction for the catch trials.

We used Mathworks® Matlab to perform the simulation. For each trial, we calculated the hand position according to equation (4) while using the estimated delay, $\tilde{\tau }$. Using the hand position data, we calculated the cursor position according to the perturbation used in the trial (Fig. 1D). To calculate the cursor position under the Delay condition we temporally shifted the hand position signal by 250ms. To calculate the cursor position under the Mechanical condition we solved the ordinary differential equation linking hand and cursor [equation (3)]. In addition, we calculated the target position signal, ${x}_{{Target}}(t)={A}_{T}\cdot \,\sin (\omega t)$, in order to calculate the tracking RMSE between it and cursor position. We repeated this procedure for all trials according to the order in Fig. 1D.

We performed the same procedure for the mechanical system based simulations. Using mechanical system based representation for the delay, the desired hand trajectory can be calculated using the estimation of the mechanical system connecting the hand and cursor positions. In this case, the system tries to estimate the two ratios between mechanical constants, $\frac{\tilde{B}}{\tilde{K}}$ and $\frac{\tilde{M}}{\tilde{K}}$ (Fig. 1C), while the desired hand position is calculated according to:

5 $${x}_{Hand}(t)={x}_{Cursor}^{Desired}(t)+\frac{\tilde{B}}{\tilde{K}}\cdot {\dot{x}}_{Cursor}^{Desired}(t)+\frac{\tilde{M}}{\tilde{K}}\cdot {\ddot{x}}_{Cursor}^{Desired}(t)$$

For the case of exact tracking, the two ratios are equal to zero. When encountering the Delay or Mechanical conditions, the value of the two ratios changes in such way that the hand trajectory will generate the desired cursor trajectory. Since the estimated spring value appears in both ratios, we can simulate the changes in these two values by changing the value of the estimated spring. To do so we set the change in the estimated spring value as an exponential function between consecutive trials, $\tilde{K}={b}_{1}+{b}_{2}\cdot \exp (i/{b}_{3})$. Similar to the estimated delay function, we found the optimal value for the constants *b*_1_, *b*_2_, *b*_3_ and the optimal estimations $\tilde{B}$ and $\tilde{M}$ separately for groups 1 and 2 so each simulation’s tracking RMSE pattern for the dominant condition would be as identical as possible to the mean RMSE pattern of each group. The optimization of the parameters did not include the catch trials. Using the optimal values we simulated the entire experiment protocol by introducing catch trials according to the experimental protocol of each group (Fig. 1D). We tested the model’s RMSE prediction for the catch trials.

Simulation procedure included calculating the hand position according to the mechanical system based representation. In each trial, we used equation (5) in order to calculate the hand position while using the estimated stiffness, $\tilde{K}$. The rest of the simulation steps were similar to the steps we used in the simulation using delayed-state representation.

### Kinematic Performance during Delay and Mechanical Conditions

Here, we tested if the difficulty to complete the task while manipulating the cursor of the mechanical system is higher than with the delayed cursor in terms of movement amplitude and movement speed required from the participants.

We estimated the expected distance between the two conditions by simulating hand movement under each condition assuming ideal tracking, i.e. the cursor was aligned perfectly with the target.

For the Delay condition, the cursor was delayed after the hand; therefore, the hand motion was simulated as:

6 $${x}_{Hand}^{D}(t)={x}_{Cursor}(t+\tau )={A}_{T}\,\sin (\omega t+\omega \tau )$$

As can be seen above, the hand should move at the same amplitude as the target, which does not depend on the tracking frequency or the time delay used.

Generally, we can calculate the transfer function between hand position and cursor position for the Delay condition:

7 $$\frac{{X}_{Hand}^{d}(s)}{{X}_{Cursor}(s)}={e}^{\tau s}$$

Since the amplitude of the last expression is equal to 1, this means that for ideal tracking, the hand amplitude should be equal to the target amplitude while the phase between the hand and the target should be equal to $\omega \tau$.

For the Mechanical condition, the hand moves according to the second-order differential equation:

8 $${x}_{Hand}^{M}(t)={x}_{Cursor}(t)+\tau \cdot {\dot{x}}_{Cursor}(t)+\frac{{\tau }^{2}}{2}{\ddot{x}}_{Cursor}(t)$$

Using the Laplace transform, we calculated a transfer function between hand position and cursor position:

9 $$\frac{{X}_{Hand}^{M}(s)}{{X}_{Cursor}(s)}=\frac{{\tau }^{2}}{2}{s}^{2}+\tau s+1=1-\frac{{\tau }^{2}}{2}{\omega }^{2}+\tau \omega j=A+Bj$$

Based on this, we expressed the hand amplitude and phase according to cursor motion:

10 $$\begin{array}{rcl}Amp & = & {A}_{T}\sqrt{{A}^{2}+{B}^{2}}\\ Phase & = & 0+\phi =0+{\tan }^{-1}(\frac{B}{A})\end{array}$$

Substituting the general parameters of the cursor motion, the expression for hand position becomes:

11 $${x}_{Hand}^{M}(t)={A}_{T}\sqrt{1+\frac{{\tau }^{4}{\omega }^{4}}{4}}\cdot \,\sin (\omega t+{\tan }^{-1}(\frac{2\tau \omega }{2-{\tau }^{2}{\omega }^{2}}))$$

The calculated amplitude of hand position which depends on tracking frequency and delay value is greater than the target amplitude.

**Figure 9:**
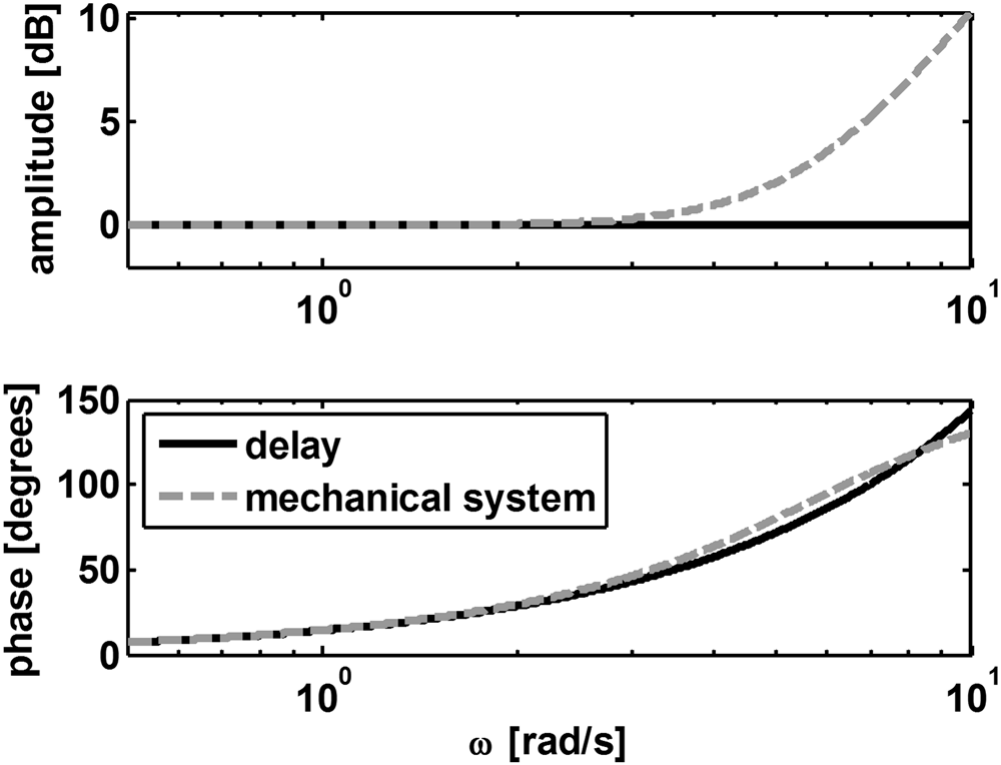
Bode plots of the transfer functions between hand position and cursor position under the Delay (solid black line) and Mechanical (gray dashed line) conditions. During the Delay condition the hand moves at the same amplitude as the cursor while during the Mechanical condition the hand amplitude increases compared to the cursor amplitude as movement frequency increases (upper panel). For both conditions the hand position leads the cursor position (bottom panel). The frequency response for the mechanical system was calculated according to the transfer function linking hand and cursor positions (equation 9) while using the delay value that was used in the experiments ($\tau =250\,ms$).

To visualize the differences in hand movement amplitude and phase between the Delay and Mechanical conditions, we used a Bode plot of the two transfer functions (equations 7 and 9) showing the desired hand amplitude for different movement frequencies (Fig. 9). We conclude that in order to track the target perfectly, the hand amplitude during the Mechanical condition needs to be larger than that during the Delay condition. Since the frequency of the position signal does not change between the two conditions, the time period remains the same for the two conditions, which means that the participants needed to move faster in the Mechanical condition.

To test whether the participants moved at larger amplitudes we extracted their movement amplitude by identifying the reversal points in the position signal. For each movement cycle we identified the local maximum and minimum points of the position signal. The absolute distance value of these reversal points were considered as the motion amplitude. We averaged these points over each trial.
